# The philosophy of scientific experimentation: a review

**DOI:** 10.1186/1759-4499-1-2

**Published:** 2009-10-29

**Authors:** Hans Radder

**Affiliations:** 1 Faculty of Philosophy, VU University Amsterdam, the Netherlands; 2 De Boelelaan 1105, 1081 HV Amsterdam, the Netherlands

## Abstract

Practicing and studying automated experimentation may benefit from philosophical reflection on experimental science in general. This paper reviews the relevant literature and discusses central issues in the philosophy of scientific experimentation. The first two sections present brief accounts of the rise of experimental science and of its philosophical study. The next sections discuss three central issues of scientific experimentation: the scientific and philosophical significance of intervention and production, the relationship between experimental science and technology, and the interactions between experimental and theoretical work. The concluding section identifies three issues for further research: the role of computing and, more specifically, automating, in experimental research, the nature of experimentation in the social and human sciences, and the significance of normative, including ethical, problems in experimental science.

## The rise of experimental science

Over the past decades the historical development of experimental science has been studied in detail. One focus has been on the nature and role of experiment during the rise of the natural sciences in the sixteenth and seventeenth centuries. Earlier accounts of this so-called Scientific Revolution emphasized the universalization of the mathematical method or the mechanization of the world-view as the decisive achievement. In contrast, the more recent studies of sixteenth and seventeenth century science stress the great significance of a new experimental practice and a new experimental knowledge. Major figures were Francis Bacon, Galileo Galilei, and Robert Boyle. The story of the controversy of the latter with Thomas Hobbes, during the late 1650s and early 1660s, has become a paradigm of the recent historiography of scientific experimentation [1]. While Hobbes defended the 'old' axiomatic-deductive style of the geometric tradition, Boyle advocated the more modest acquisition of probable knowledge of experimental 'matters of fact'. Simultaneously at stake in this controversy were the technical details of Boyle's air-pump experiments, the epistemological justification of the experimental knowledge and the social legitimacy of the new experimental style of doing science.

A more wide-ranging account of the role of experimentation in the natural sciences has been proposed by Thomas Kuhn [2]. He claims that the rise of modern physical science resulted from two simultaneous developments. On the one hand, a radical conceptual and world-view change occurred in what he calls the classical, or mathematical, sciences, such as astronomy, statics and optics. On the other, the novel type of Baconian, or experimental, sciences emerged, dealing with the study of light, heat, magnetism and electricity, among other things. Kuhn argues that it was not before the second half of the nineteenth century that a systematic interaction and merging of the experimental and mathematical traditions took place. An example is the transformation of the Baconian science of heat into an experimental-mathematical thermodynamics during the first half of the nineteenth century. At about the same time, the interactions between (at first, mainly experimental) science and technology increased substantially. Important results of this scientification of technology were chemical dye stuffs and artificial fertilizers.

Starting in the second half of the nineteenth century, extensive experimentation also took root in various other sciences. This happened in medicine, in particular in physiology, somewhat later in psychology, and still later in the social sciences. A characteristic feature of many experiments in those sciences is a strong reliance on statistical methods (see, e.g., [3]).

## The rise of the philosophy of scientific experimentation

Alongside the actual practices of experimentation, a variety of authors--both philosophers and philosophy-minded scientists--have reflected upon the nature and function of scientific experiments. Among the better-known examples are Bacon's and Galileo's advocacy of the experimental method. John Stuart Mill (around the middle of the nineteenth century) and Ernst Mach (late nineteenth-early twentieth century) provided some methodological and epistemological analyses of experimentation. Claude Bernard promoted and analyzed the use of the experimental method in medicine. His *Introduction to the Study of Experimental Medicine *[4] influenced a number of twentieth century French writers, including Pierre Duhem, Gaston Bachelard and Georges Canguilhem. While those authors addressed some aspects of experimentation in their accounts of science, a substantial and coherent tradition in the philosophy of scientific experimentation did not yet arise.

Such a tradition did spring up in Germany, in the second half of the twentieth century. Within this German tradition two approaches may be distinguished. One developed Hugo Dingler's pioneering work [5]. Dingler emphasized the manipulation and intervention character of experimentation, and hence its kinship to technology. One of his aims was to show how the basic theoretical concepts of physics, such as length or mass, could be grounded in concrete experimental actions. During the 1960s and 1970s, this part of Dingler's views was taken up and systematically developed by several other German philosophers, including Paul Lorenzen, Klaus Holzkamp and Peter Janich. More recently, the emphasis on the methodical construction of theoretical concepts in terms of experimental actions has given way to a more culturalistic interpretation of experimental procedures and results [6].

A second approach within the German tradition took its departure even more directly from the kinship between experiment and technology. The major figure here is the early Jürgen Habermas. In his work from the 1960s, Habermas conceived of (empirical-analytical) science as 'anticipated technology', the crucial link being experimental action [7]. In the spirit of Karl Marx, Martin Heidegger and Herbert Marcuse, Habermas' aim was not merely to develop a theory of (scientific) knowledge but rather a critique of technocratic reason. More recently, attempts have been made to connect this German tradition to Anglo-Saxon philosophy of experiment [8,9] and to contemporary social studies of science and technology [10]. Recent work on 'science as technology' by Srđan Lelas [11] can be characterized as, broadly, inspired by this second branch of the German tradition.

In the English-speaking world, a substantial number of studies of scientific experimentation have been written since the mid-1970s. They resulted from the Kuhnian 'programs in history and philosophy of science'. In their studies of (historical or contemporary) scientific controversies, sociologists of scientific knowledge often focused on experimental work (e.g., [12]), while so-called laboratory studies addressed the ordinary practices of experimental scientists (e.g., [13]). An approach that remained more faithful to the history and philosophy of science idea started with Ian Hacking's argument for the relative autonomy of experimentation and his plea for a philosophical study of experiment as a topic in its own right [14]. It includes work by Allan Franklin, Peter Galison, David Gooding and Hans-Jörg Rheinberger, among many others (see the edited volumes [15,16] and [17]).

More recently, several philosophers argue that a further step should be taken by combining the results of the historical and sociological study of experiment with more developed theoretical-philosophical analyses [18]. A mature philosophy of experiment, they claim, should not be limited to summing up its practical features but attempt to provide a systematic analysis of experimental practice and experimental knowledge. The latter is often lacking in the sociological and historical literature on scientific experimentation.

## Intervention and production, and their philosophical implications

Looking at the specific features of experiments within the overall practice of science, there is one feature that stands out. In order to perform experiments, whether they are large-scale or small-scale, experimenters have to *intervene *actively in the material world; moreover, in doing so they *produce *all kinds of new objects, substances, phenomena and processes. More precisely, experimentation involves the material realization of the experimental system (that is to say, the object(s) of study, the apparatus, and their interaction) as well as an active intervention in the environment of this system. In this respect, experiment contrasts with theory even if theoretical work is always attended with material acts (such as the typing or writing down of a mathematical formula). Hence, a central issue for a philosophy of experiment is the question of the nature of experimental intervention and production, and their philosophical implications. To be sure, at times scientists devise and discuss so-called thought experiments [19]. However, such 'experiments'--in which the crucial aspect of intervention and production is missing--are better conceived as not being experiments at all but rather as particular types of theoretical argument, which may or may not be materially realizable in experimental practice.

Clearly, not just any kind of intervention in the material world counts as a scientific experiment. Quite generally, one may say that successful experiments require, at least, a certain stability and reproducibility, and meeting this requirement presupposes a measure of control of the experimental system and its environment as well as a measure of discipline of the experimenters and the other people involved in realizing the experiment.

Experimenters employ a variety of strategies for producing stable and reproducible experiments (see, e.g., [20,21] and [6]). One such strategy is to attempt to realize 'pure cases' of experimental effects. For example, in some early electromagnetic experiments carried out in the 1820s, André Ampère investigated the interaction between an electric current and a freely suspended magnetic needle [22]. He systematically varied a number of factors of his experimental system and examined whether or not they were relevant, that is to say, whether or not they had a destabilizing impact on the experimental process.

Furthermore, realizing a stable object-apparatus system requires knowledge and control of the (actual and potential) interactions between this system and its environment. Depending on the aim and design of the experiment, specific interactions may be *necessary *(and hence required), *allowed *(but irrelevant), or *forbidden *(because disturbing). Thus, in his experiments on electromagnetism, Ampère anticipated a potential disturbance exerted by the magnetism of the earth. In response, he designed his experiment in such a way that terrestrial magnetism constituted an allowed rather than a forbidden interaction.

A further aspect of experimental stability is implied by the notion of reproducibility [9]. A successful performance of an experiment by the original experimenter is an achievement that may depend on certain idiosyncratic aspects of a local situation. Yet, a purely local experiment that cannot be carried out in other experimental contexts will, in the end, be unproductive for science. However, since the performance of an experiment is a complex process, no repetition will be strictly identical to the original experiment and many repetitions may be dissimilar in several respects. For this reason, we need to specify *what *we take or require to be reproducible (for instance, a particular aspect of the experimental process or a certain average over different runs). Furthermore, there is the question of *who *should be able to reproduce the experiment (for instance, the original experimenter, contemporary scientists, or even any scientist or human being). Investigating these questions leads to different types and ranges of experimental reproducibility, which can be observed to play different roles in experimental practice.

Laboratory experiments in physics, chemistry and molecular biology often allow one to control the objects under investigation to such an extent that the relevant objects in successive experiments may be assumed to be in identical states. Hence, statistical methods are employed primarily to further analyze or process the data (see, for instance, the error-statistical approach by Deborah Mayo [23]). In contrast, in field biology, medicine, psychology and social science, such a strict experimental control is often not feasible. To compensate for this, statistical methods in these areas are used directly to construct groups of experimental subjects that are presumed to possess identical average characteristics. It is only after such groups have been constructed that one can start the investigation of hypotheses about the research subjects. One can phrase this contrast in a different way by saying that in the former group of sciences statistical considerations mostly bear upon linking experimental data and theoretical hypotheses, while in the latter group it is often the case that statistics already play a role at the stage of producing the actual individual data.

The intervention and production aspect of scientific experimentation carries implications for several philosophical questions. A general lesson, already drawn by Bachelard, appears to be this: the intervention and production character of experimentation entails that the actual objects and phenomena themselves are, at least in part, materially realized through human interference. Hence, it is not just the knowledge of experimental objects and phenomena but also their actual existence and occurrence that prove to be dependent on specific, productive interventions by the experimenters. This fact gives rise to a number of important philosophical issues. If experimental objects and phenomena have to be realized through active human intervention, does it still make sense to speak of a 'natural' nature or does one merely deal with artificially produced laboratory worlds? If one does not want to endorse a fully-fledged constructivism, according to which the experimental objects and phenomena are nothing but artificial, human creations, one needs to develop a more differentiated categorization of reality. In this spirit, various authors (e.g., [20,9]) have argued that an appropriate interpretation of experimental science needs some kind of dispositional concepts, such as powers, potentialities, or tendencies. These human-independent dispositions would then underlie and enable the human construction of particular experimental processes.

A further important question is whether scientists, on the basis of artificial experimental intervention, can acquire knowledge of a human-independent nature. Some philosophers claim that, at least in a number of philosophically significant cases, such 'back inferences' from the artificial laboratory experiments to their natural counterparts can be justified. Another approach accepts the constructed nature of much experimental science, but stresses the fact that its results acquire a certain endurance and autonomy with respect to both the context in which they have been realized in the first place and later developments. In this vein, Davis Baird [24] offers an account of 'objective thing knowledge', the knowledge encapsulated in material things, such as Watson and Crick's material double helix model or the Indicator of Watt and Southern's steam engine.

Another relevant feature of experimental science is the distinction between the working of an apparatus and its theoretical accounts. In actual practice it is often the case that experimental devices work well, even if scientists disagree on how they work. This fact supports the claim that variety and variability at the theoretical level may well go together with a considerable stability at the level of the material realization of experiments. This claim can then be exploited for philosophical purposes, for example to vindicate entity realism [14] or referential realism [8].

## The relationship between (experimental) science and technology

Traditionally, philosophers of science have defined the aim of science as, roughly, the generation of reliable knowledge of the world. Moreover, as a consequence of explicit or implicit empiricist influences, there has been a strong tendency to take the production of experimental knowledge for granted and to focus on theoretical knowledge. However, if one takes a more empirical look at the sciences, both at their historical development and at their current condition, this approach must be qualified as one-sided. After all, from Archimedes' lever-and-pulley systems to the cloned sheep Dolly, the development of (experimental) science has been intricately interwoven with the development of technology ([25,26]). Experiments make essential use of (often specifically designed) technological devices, and, conversely, experimental research often contributes to technological innovations. Moreover, there are substantial conceptual similarities between the realization of experimental and that of technological processes, most significantly the implied possibility and necessity of the manipulation and control of nature. Taken together, these facts justify the claim that the science-technology relationship ought to be a central topic for the study of scientific experimentation.

One obvious way to study the role of technology in science is to focus on the instruments and equipment employed in experimental practice. Many studies have shown that the investigation of scientific instruments is a rich source of insights for a philosophy of scientific experimentation (see, e.g. [15,17,18] and [27]). One may, for example, focus on the role of visual images in experimental design and explore the wider problem of the relationship between thought and vision. Or one may investigate the problem of how the cognitive function of an intended experiment can be materially realized, and what this implies for the relationship between technological functions and material structures. Or one may study the modes of representation of instrumentally mediated experimental outcomes and discuss the question of the epistemic or social appraisal of qualitative versus quantitative results.

In addition to such studies, several authors have proposed classifications of scientific instruments or apparatus. One suggested distinction is that between instruments that represent a property by measuring its value (e.g., a device that registers blood pressure), instruments that create phenomena that do not exist in nature (e.g., a laser), and instruments that closely imitate natural processes in the laboratory (e.g., an Atwood machine, which mimics processes and properties of falling objects).

Such classifications form an excellent starting point for investigating further philosophical questions on the nature and function of scientific instrumentation. They demonstrate, for example, the inadequacy of the empiricist view of instruments as mere enhancers of human sensory capacities. Yet, an exclusive focus on the instruments as such may tend to ignore two things. First, an experimental setup often includes various 'devices', such as a concrete wall to shield off dangerous radiation, a support to hold a thermometer, a spoon to stir a liquid, curtains to darken a room, and so on. Such devices are usually not called instruments, but they are equally crucial to a successful performance and interpretation of the experiment and hence should be taken into account. Second, a strong emphasis on instruments may lead to a neglect of the environment of the experimental system, especially of the requirement to control the interactions between the experimental system and its environment. Thus, a comprehensive view of scientific experimentation needs to go beyond an analysis of the instrument as such by taking full account of the specific setting in which this instrument needs to function.

Finally, there is the issue of the general philosophical significance of the experiment-technology relationship. Some of the philosophers who emphasize the importance of technology for science endorse a 'science-as-technology' account. That is to say, they advocate an overall interpretation in which the nature of science--not just experimental but also theoretical science--is seen as basically or primarily technological (see for instance, [5,7] and [11]). Other authors, however, take a less radical view by criticizing the implied reduction of science to technology and by arguing for the *sui generis *character of theoretical-conceptual and formal-mathematical work. Thus, while stressing the significance of the technological--or perhaps, more precisely, the intervention and production dimension of science--these views nevertheless see this dimension as complementary to a theoretical dimension (see, e.g., [8,24] and [28]).

## The role of theory in experimentation

This brings us to a further central theme in the study of scientific experimentation, namely the relationship between experiment and theory. The theme can be approached in two ways. One approach addresses the question of how theories or theoretical knowledge may arise from experimental practices. Thus, Franklin [21] has provided detailed descriptions and analyses of experimental confirmations and refutations of theories in twentieth century physics. Giora Hon [28] has put forward a classification of experimental error, and has argued that the notion of error may be exploited to elucidate the transition from the material, experimental processes to propositional, theoretical knowledge (see also [29]).

A second approach to the experiment-theory relationship examines the question of the role of existing theories, or theoretical knowledge, within experimental practices. Over the last 25 years, this question has been debated in detail. Are experiments, factually or logically, dependent on prior theories, and if so, in which respects and to what extent? The remainder of this section reviews some of the debates on this question.

The strongest version of the claim that experimentation is theory dependent says that all experiments are planned, designed, performed, and used from the perspective of one or more theories about the objects under investigation. In this spirit, Justus von Liebig and Karl Popper, among others, advocated the view that all experiments are explicit tests of existing theories. This view completely subordinates experimental research to theoretical inquiry. However, on the basis of many studies of experimentation published during the last 25 years, it can be safely concluded that this claim is false. For one thing, quite frequently the aim of experiments is just to realize a stable phenomenon or a working device. Yet, the fact that experimentation involves much more than theory testing does not, of course, mean that testing a theory may not be an important goal in particular scientific settings.

At the other extreme, there is the claim that, basically, experimentation is theory-free. The older German school of 'methodical constructivism' (see [6]) came close to this position. A somewhat more moderate view is that, in important cases, theory-free experiments are possible and do occur in scientific practice. This view admits that performing such 'exploratory' experiments does require some ideas about nature and apparatus, but not a well-developed theory about the phenomena under scrutiny. Ian Hacking [14] and Friedrich Steinle [22] make this claim primarily on the basis of case studies from the history of experimental science. Michael Heidelberger [30] aims at a more systematic underpinning of this view. He distinguishes between theory-laden and causally-based instruments and claims that experiments employing the latter type of instruments are basically theory-free.

Another view admits that not all concrete activities that can be observed in scientific practice are guided by theories. Yet, according to this view, if certain activities are to count as a genuine experiment, they require a theoretical interpretation (see [8,9,28] and [31]). More specifically, performing and understanding an experiment depends on a theoretical interpretation of what happens in materially realizing the experimental process. In general, quite different kinds of theory may be involved, such as general background theories, theories or theoretical models of the (material, mathematical, or computational) instruments, and theories or theoretical models of the phenomena under investigation.

One argument for such claims derives from the fact that an experiment aims to realize a reproducible correlation between an observable feature of the apparatus and a feature of the object under investigation. The point is that materially realizing this correlation and knowing what can be learned about the object from inspecting the apparatus depends on theoretical insights about the experimental system and its environment. Thus, these insights pertain to those aspects of the experiment that are relevant to obtaining a reproducible correlation. It is not necessary, and in practice it will usually not be the case, that the theoretical interpretation offers a full understanding of any detail of the experimental process.

A further argument for the significance of theory in experimentation notes that a single experimental run is not enough to establish a stable result. A set of different runs, however, will almost always produce values that are, more or less, variable. The questions then are: What does this fact tell us about the nature of the property that has been measured? Does the property vary within the fixed interval? Is it a probabilistic property? Or is its real value constant and are the variations due to random fluctuations? In experimental practice, answers to such questions are based on an antecedent theoretical interpretation of the nature of the property that has been measured.

Regarding these claims, it is important to note that, in actual practice, the theoretical interpretation of an experiment will not always be explicit and the experimenters will not always be aware of its use and significance. Once the performance of a particular experiment or experimental procedure becomes routine, the theoretical assumptions drop out of sight: they become like an (invisible) 'window to the world'. Yet, in a context of learning to perform and understand the experiment or in a situation where its result is very consequential or controversial, the implicit interpretation will be made explicit and subjected to empirical and theoretical scrutiny. This means that the primary locus of the theoretical interpretation is the relevant scientific community and not the individual experimenter.

## In conclusion: further issues in scientific experimentation

As we have seen, the systematic philosophical study of scientific experimentation is a relatively recent phenomenon. Hence, there are a number of further issues that have received some attention but merit a much more detailed account. In concluding this review paper, three such issues will be briefly discussed.

First, recent scientific practice shows an ever-increasing use of 'computer experiments'. These involve various sorts of hybrids of material intervention, computer simulation, and theoretical and mathematical modeling techniques (see [32]). Often, more traditional experimental approaches are challenged and replaced by approaches resting fully or primarily on computer simulations (sometimes this replacement is based on budgetary considerations only). More generally, there is a large variety of uses of computer science and technology in performing, analyzing and interpreting experiments and in visualizing, storing and disseminating their results. Automated experimentation constitutes a significant part of these developments.

These new developments raise important questions for the scholarly study of scientific experimentation. First, although some pioneering work has been done (see, for instance, [33] about the role of databases, and, more generally, bioinformatics in research in the life sciences), we need many more empirical studies that chart this new terrain. Furthermore, new methodological questions arise about how to do this automated experimentation in innovative, yet plausible, ways. As the history of Artificial Intelligence teaches us, expectations about automation can sometimes be overenthusiastic and unfounded ([34,35]). For this reason, a critical assessment of what can, and what cannot, be achieved through automation is particularly important (for the cases of formal symbol manipulation and neural network approaches to AI, see [36], chaps. 5 and 12). Related to this is the epistemological question of the justifiability of the results of the new approaches. Should experiments always involve a substantial material component or are simulated experiments equally reliable and useful (see [37])? Finally, computer experiments are regularly applied to complex and large-scale systems, for instance in climate science. Often, in such contexts, scientific and policy problems are intimately connected. This connection also constitutes an important topic for the study of scientific experimentation (see, e.g., [38]).

A second issue that merits more attention is the nature and role of experimentation in the social and human sciences, such as economics, sociology, medicine, and psychology. Practitioners of those sciences often label substantial, or even large, parts of their activities as 'experimental'. So far, this fact is not reflected in the philosophical literature on experimentation, which has primarily focused on the natural sciences. Thus, a challenge for future research is to connect the primarily methodological literature on experimenting in economics, sociology, medicine, and psychology with the philosophy of science literature on experimentation in natural science (see, e.g., [39] and [40]).

One subject that will naturally arise in philosophical reflection upon the similarities and dissimilarities of natural and social or human sciences is this: In experiments on human beings, the experimental subjects will often have their own interpretation of what is going on in these trials, and this interpretation may influence their responses over and above the behavior intended by the experimenters. As a methodological problem (of how to avoid 'biased' responses) this is of course well known to practitioners of the human and social sciences. However, from a broader philosophical or socio-cultural perspective the problem is not necessarily one of bias. It may also reflect a clash between a scientific and a common-sense interpretation of human beings. In case of such a clash, social and ethical issues are at stake, since the basic question is who is entitled to define the nature of human beings: the scientists or the people themselves? The methodological, ethical, and social issues springing from this question will continue to be a significant theme for the study of experimentation in the human and social sciences.

This brings us to a last issue. The older German tradition explicitly addressed wider normative questions surrounding experimental science and technology. The views of Habermas, for example, have had a big impact on broader conceptualizations of the position of science and technology in society. Thus far, the more recent Anglophone approaches within the philosophy of scientific experimentation have primarily dealt with more narrowly circumscribed scholarly topics. In so far as normative questions have been taken into account, they have been mostly limited to epistemic normativity, for instance to questions of the proper functioning of instruments or the justification of experimental evidence. Questions regarding the connections between epistemic and social or ethical normativity are hardly addressed.

Yet, posing such questions is not far-fetched. For instance, those experiments that use animals or humans as experimental subjects are confronted with a variety of normative issues, often in the form of a tension between methodological and ethical requirements [41]. Other normatively relevant questions relate to the issue of the artificial and the natural in experimental science and science-based technology. Consider, for example, the question of whether experimentally isolated genes are natural or artificial entities. This question is often discussed in environmental philosophy, and different answers to it entail a different environmental ethics and politics. More specifically, the issue of the contrast between the artificial and the natural is crucial to debates about patenting, in particular the patenting of genes and other parts of organisms. The reason is that discoveries of natural phenomena are not patentable while inventions of artificial phenomena are [42].

Although philosophers of experiment cannot be expected to solve all of those broader social and normative problems, they may be legitimately asked to contribute to the debate on possible approaches and solutions. In this respect, the philosophy of scientific experimentation could profit from its kinship to the philosophy of technology, which has always shown a keen sensitivity to the interconnectedness between technological and social or normative issues.

## Competing interests

The author declares that he has no competing interests.
